# HIVE MIND - Establishing diagnostic criteria for HIV associated acquired epidermodysplasia verruciformis: an international Delphi study.

**DOI:** 10.12688/wellcomeopenres.26218.3

**Published:** 2026-08-29

**Authors:** Sophie H Kelly, Stephen L Walker, Nicola Willis, Rashida A Ferrand, L Claire Fuller, Eneyi Kpokiri, Vimbainashe Karimatsenga, Sinéad M Langan

**Affiliations:** 1 London School of Hygiene & Tropical Medicine, London, England, UK; 2 Biomedical Research and Training Institute, Harare, Harare Province, Zimbabwe; 3 Zvandiri, Harare, Zimbabwe; 4 Chelsea and Westminster Hospital NHS Foundation Trust, London, England, UK; 5 University of Zimbabwe, Harare, Harare Province, Zimbabwe

**Keywords:** HIV, Epidermodysplasia Verruciformis, Acquired Epidermodysplasia Verruciformis, Flat warts, Diagnostic criteria, Delphi

## Abstract

**Background:**

Acquired epidermodysplasia verruciformis (aEV) is a skin condition caused by increased vulnerability to cutaneous human papillomaviruses. aEV disproportionately affects people who acquire HIV perinatally. There are no accepted diagnostic criteria for aEV. The lack of routine diagnosis has limited research into this condition. There are no community prevalence estimates, no evidence-based treatments and no cohort studies to understand the risk of skin malignancy. Most people with perinatally-acquired HIV, live in low-resource settings without access to specialist skin doctors or skin biopsies. There is therefore a need to develop diagnostic criteria that can be used by non-specialists and are applicable in low-resource settings.

**Aim:**

This Delphi study aims reach consensus on screening and diagnostic criteria for HIV-associated aEV which are applicable in both high and low resource settings.

**Methods:**

Modified Delphi methodology will be used to achieve expert consensus. A panel of 20–30 experts with at least 5 years’ experience in HIV or dermatology and experience with aEV will be asked to complete a series of surveys. Firstly, they will contribute all possible features of aEV. They will then rate these features in terms of importance. This will be used to guide the creation of draft diagnostic criteria. Panellists will rate their agreement with each criterion and adoption of the criteria as a whole. This will continue until consensus is reached. Consensus is defined a priori as when more than >60% of the panel either agree or strongly agree with each criterion and ≥ 80% of the panel with the adoption of criteria as a whole.

**Expected Outcomes:**

A screening tool to identify people with possible aEV who warrant further examination.

A set of diagnostic criteria that can be applied in real world settings to diagnose aEV.

**Ethical Approval:**

This study has been approved by the LSHTM Research Ethics Committee (Reference: 33189.)

## Plain Language summary

Acquired epidermodysplasia verruciformis (aEV) is a skin condition caused by an increased vulnerability to human papillomaviruses on the skin, similar to the viruses that cause common warts. This skin condition can occur in people who have a weakened immune system due to HIV. There is very little information about the number of people living with HIV-associated aEV across the world. This is, in part, because there is no globally accepted way to diagnose this condition. The aim of this study is to gather experts to agree on the best way to diagnose HIV-associated aEV in both high and low resource settings.

The study uses a method known as a Delphi process. This consists of recruiting experts in the field to a panel. In this case doctors, nurses and health care professionals with over five years’ experience and experience of looking after people with aEV will be recruited. They will be asked to complete a series of surveys. In the first survey they will be asked to contribute all possible features of aEV. In the second they will be asked to rate each feature in terms of importance. These results will be collated by the study team and used to produce draft diagnostic criteria. The panellists will be asked to review these criteria, rate their agreement and provide comments. These answers will be used to revise the criteria, and this process will continue until over 60% of the panel agree with each aspect of the criteria and 80% agree with adoption of the criteria as a whole.

At the end of this study the first diagnostic criteria for aEV will be shared. These can be used for diagnosis in the clinic and to recruit people with aEV to research studies.

## Introduction

Acquired epidermodysplasia verruciformis (aEV) was a term coined in 2009 to describe a dermatological condition caused by increased susceptibility to cutaneous human papillomaviruses (HPV) in the context of immunosuppression. ^1^ The histopathology and clinical presentation are identical to the genodermatosis epidermodysplasia verruciformis (EV) in which the increased susceptibility to cutaneous HPV is caused by well described mutations two genes EVER1/TMC6 and EVER2/TMC8. ^1– 3^

aEV can occur in the context of T-cell immunosuppression, it has been observed to occur in people living with HIV, particularly in those who acquired HIV perinatally. ^4– 7^ Unlike the congenital form of the condition which has a clear genetic test, aEV has no single diagnostic test. ^1^ Diagnosis is typically made based on a combination of history, clinical features, and, if available, typical features on skin biopsy. In the research context, HPV genotyping is used to confirm the presence of beta-HPV types. ^1^^,^^4, 8^ This is not done routinely in a clinical setting. ^4– 7^

The exact mechanism of development of aEV in people with HIV is not fully elucidated. There is evidence that immune reconstitution in the skin compartment is not complete in those who are diagnosed with a CD4 ≤350 cells/mm ^3^. ^9^ Whilst this alone does not explain the disproportionate number of cases observed in people with perinatally acquired HIV, a hypothesis is that a poor T cell response upon first exposure to beta-HPVs as an infant or child increases the risk of beta-HPV persistence and aEV development. ^9^ ^–^ ^11^

Clinical and scientific knowledge of HIV-associated aEV is predominantly based on descriptions of patients in case report and case series, small cohort studies and two systematic reviews. ^8, 12^ Sixty percent (n=48) of the 80 reported cases in the systematic review by Cuestas et al., were in people from Africa. ^8^ The remainder of the cases were from the Americas (n = 17), Europe (n=14) and Asia (n=1). ^8^ The described clinical presentation was predominantly of widespread flat warts, hypopigmented macules and pityriasis versicolour-like lesions across the face upper limbs and upper trunk. ^8, 12^ There was no standard method of diagnosis, with biopsy carried out in 37.5% of cases and HPV typing carried out in 73.5%; the most detected genotypes were 5, 20 and 8. ^8^

There are no good prevalence estimates of the global distribution of HIV-associated aEV. This is in part due to the lack of clear clinical diagnostic criteria. The majority of people living with HIV, and perinatally acquired HIV live in the World Health Organisation (WHO) Africa Region, most cases of HIV-associated aEV cases occur in settings where there is limited access to histopathology, dermatological training. Diagnosis, if made, is primarily clinical. ^4^ ^,^ ^6^ ^,^ ^8^

The congenital form of EV is associated with progression to skin malignancy in the 3 ^rd^ or 4 ^th^ decade of life. ^13^ These patients are advised to have regular skin checks and early investigation of suspicious lesions. ^14^ This is not routine practice for patients with HIV-associated aEV in the Global South and, although some cases have been reported, the rate of progression to malignancy is not known. ^8^ ^,^^15^^–^ ^18^

In May 2025, at the 78 ^th^ World Health Assembly member states adopted a resolution titled “Skin Diseases as a Global Public Health Priority”. ^19^ As part of this resolution, member states committed to enhance data collection and mapping of skin disease and to promote research on skin disease. ^19^ The variation in diagnostics limits the research and characterisation of the prevalence of aEV. The aim of this study is to develop consensus diagnostic criteria which will be essential for epidemiological mapping, recruitment to further research studies, for example to quantify malignancy risk in this population and referral for clinical services.

## Study rationale

There are no agreed diagnostic criteria for aEV. There is no consensus on whether histopathology is necessary for diagnosis or whether a clinical diagnosis is sufficient. This limits recruitment to and comparison of cohort studies and reporting of key outcomes, such as the risk of skin cancer, in this group. Diagnostic criteria are essential for accurate epidemiological mapping, research studies, in addition to referral for clinical services.

This study will use Delphi methodology to achieve consensus for diagnostic criteria. ^20^ Delphi methodology relies on the formation of a group of expert panellists who are questioned on the issue of interest. ^21– 23^ Collated responses to each round of the survey are sent to the panellists and used to inform the next round. Their responses are anonymous to avoid social pressure and conformity bias. ^21, 22^

Use of a Delphi technique is useful in this context given that aEV is rare and there is limited empirical data from observational studies. The Delphi technique has been used successfully to develop diagnostic criteria for other rare skin conditions. ^24, 25^ The Delphi study is overseen by a steering committee, who integrate participant responses with a review of the literature to inform round development.

An online Delphi allows the recruitment of experts from across the globe and gives panellists more flexibility in offering their opinions. ^24^ The Delphi technique preserves anonymity between panellists allowing them to express their opinions and minimising the impact of one influential voice. This is important given the relative scarcity of experts in this area.

We aim to recruit an inclusive panel consisting of nurses, doctors and clinical officers from at least four WHO regions and with real-world experience of diagnosing HIV-associated aEV. This ensures we develop diagnostic criteria that are relevant, academically sound and directly applicable in both high and low resource settings. The design of the study has been discussed with a community advisory board consisting of members with lived experience of skin disease and HIV. These colleagues will advise at key points throughout the study to ensure real-world acceptability and applicability. ^26^

## Study objectives and aims

Aim: To develop screening and diagnostic criteria for HIV-associated aEV that are applicable in both high- and low-resource settings.

Key definitions and responsibilities.

**Steering Committee:** The project will be overseen by a steering committee consisting of a dermatologists, HIV clinicians and HIV nurses. The role of the steering committee will be to review study results, collate feedback, draft appropriate diagnostic criteria for the expert panel to review and to decide when the study has met consensus. The steering committee will meet every 4 weeks for the duration of the Delphi study and will liaise closely with the community advisory board (CAB).

**Expert Panel:** The expert panellists are the Delphi participants and will be responsible for responding to the surveys and emails sent by the facilitator (study PI).

**Facilitator:** The facilitator is the study PI. They will be responsible for all communication with the expert panel, co-ordinating and leading the meetings with the steering committee and communicating with the CAB.

**Community advisory board:** The CAB is an independent group of people living with HIV and aEV. The members of the CAB are colleagues in the study and are well placed to advise on the acceptability and usability of any potential criteria. They will be consulted at all stages of the study and have been involved in design of the protocol. The study facilitator will liaisie with the CAB at key points throughout the study, including in the design phase, to present draft diagnostic criteria and expert feedback and in the dissemination phase. The study facilitator will liaise closely with the CAB to ensure inclusivity and that the final diagnostic criteria are acceptable to people living with aEV. If and when necessary, the CAB will be invited to present their discussions to the steering committee and vice versa.

## Study protocol

### Study design

The Delphi will be hosted virtually, and the panellists will fill in questions on online forms. The questions in the first round will be open-ended and exploratory and in the later rounds will be confirmatory. The study will continue until the threshold for consensus is met.

The virtually hosted Delphi has been selected for flexibility; panellists can fill in the forms in their own time around their schedules with no requirement for synchronous discussion across different time zones. This modification preserves the core principles of Delphi methodology and allows inclusion of people from many different settings with busy schedules.

### Study population

The target population for the expert panel are dermatologists, HIV clinicians, nurses and dermatology clinical officers with five or more years’ experience **and** experience of managing patients living with aEV. Five years has been chosen to ensure that all panellists have sufficient expertise, whilst allowing more junior colleagues with relevant expertise to participate. To reflect the need for internationally applicable diagnostic criteria, recruitment will focus on members from all WHO regions; with a particular drive to increase participation from the WHO Africa region. This region has a disproportionate burden of HIV and aEV but is under-represented in the current literature. We will therefore only close recruitment when at least 15 participants from the WHO Africa region have joined the panel.

**Inclusion criteria:**

• Qualified nurse, doctor, or clinical officer with five or more years’ experience in managing patients with skin disease **or** HIV
• Experience managing people with aEV

**Exclusion criteria:**

• Member of the steering committee
• Unable to commit time to Delphi panel (approximately 20 minutes month for the duration of the study)
• Other conflict of interest

### Recruitment strategy

**Figure 1.**
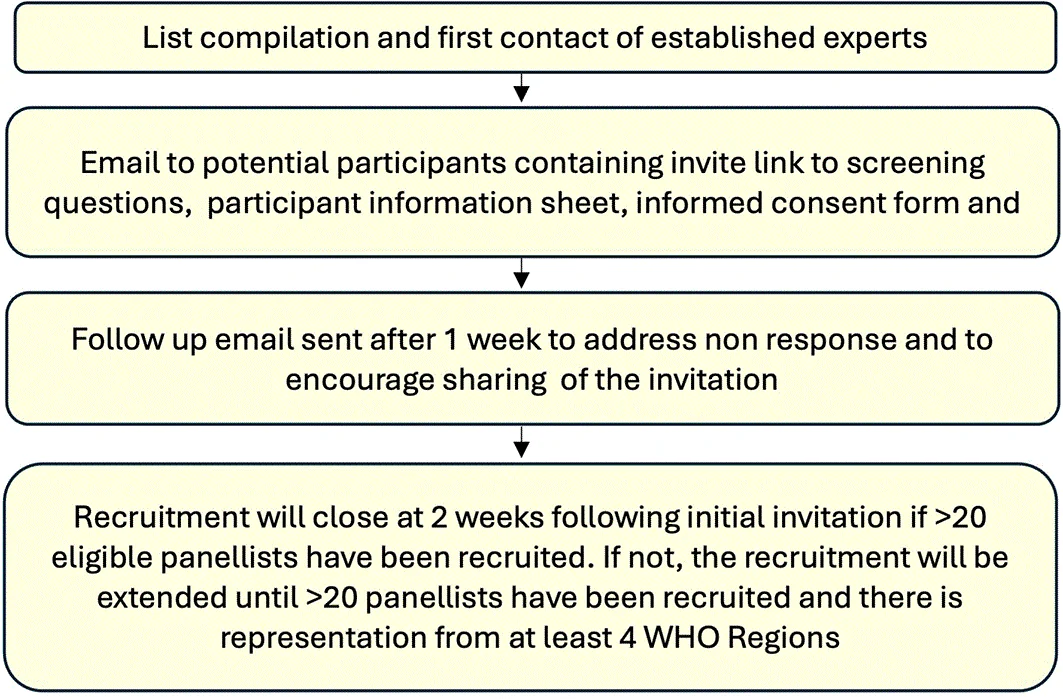
Expert panel recruitment process.

The recruitment strategy is shown in Figure 1. A pool of initial candidates will be drawn up using:

1. **Targeted purposive sampling**: The study team will contact established experts in the field of HIV and dermatology inviting them to participate in the study.
2. **Snowball sampling**: The invited experts from step 1, will be asked to share the invitation widely with their networks.
3. **Literature Review**: Published case reports, case series and systematic reviews from the last 15 years will be identified using a search of PubMed using the terms “epidermodysplasia verruciformis”. A list of corresponding and first authors will be made and screened for inclusion.

### Sample size

The planned panel size is 20–30 panellists. This size is intentionally moderate, reflecting both the scarcity of experts in HIV-associated aEV and the methodological goal of the study. ^27^ Since our panel represents a group of highly specialised clinicians, we are prioritising depth of experience over stakeholder diversity. ^27^ Panellists will be selected based on objective criteria, specifically requiring a minimum of 5 years of clinical experience and experience with aEV.

## Study procedures

### Literature review

The study facilitator will search PubMed for all papers using broad search terms for epidermodysplasia verruciformis AND HIV. Abstracts will be reviewed with the inclusion and exclusion criteria below. Full texts will be screened for all relevant abstracts. The clinical features, biopsy findings and virology of all described cases will be extracted using the schema presented below. The variables to be extracted from each paper are available in the extended data (Appendix 1). ^28^

**Inclusion criteria:**

• Clinical trial, cross-sectional, cohort study, case report or case series describing at least one of clinical OR biopsy OR virological findings in people with acquired EV AND HIV

**Exclusion criteria:**

• Unable to disaggregate the data from those with other underlying causes of immunosuppression
• Unable to access full text

A summary of this literature review will be presented to the steering committee and will be used to inform the diagnostic criteria presented for review.

The literature review will not be circulated to the participants prior to round 1 to avoid influencing their opinions. The majority of cases reported in the literature come from case reports in high resource settings, where presentation and access to tests may be different from the experience of our panellists. The diagnostic criteria developed using this Delphi are intended to be based on the clinical experience of clinicians who see this condition regularly and not unduly influenced by cases presented in case reports.

### Instrument development and piloting

The surveys will be conducted using REDCap hosted by the London School of Hygiene and Tropical Medicine. ^29^ There is capacity to save the survey and return, if necessary, this allows participants to split the survey into manageable chunks.

The surveys for each round will be developed by SK in discussion with the steering committee and a test version piloted by members of the steering committee. Any issues at this stage will be rectified before publishing the surveys.

### Screening question and panel selection

Potential candidates will be emailed an invitation to an online questionnaire and asked to share this with their networks. The first page of the questionnaire asks potential participants whether they are a nurse, doctor or clinical officer with 5 or more years’ experience in HIV or dermatology and whether they have experience managing patients with aEV. If they answer no to either of these questions the survey is terminated and no data is collected.

Those that meet the inclusion criteria will be directed to read the participant information sheet and sign an electronic consent form. Participants will be able to submit an e-signature and using the REDCap e-consent tool, they will be able to review, download a pdf copy and confirm their responses before submitting. ^30^ This is considered equivalent to written consent. ^30^

They will then proceed to a survey on their demographic characteristics and experience. The recruitment period may be extended to ensure a diversity of panellists, at least one panellist from four WHO regions and at least 15 panellists from the African region.

### Flow of Delphi rounds

**Figure 2.**
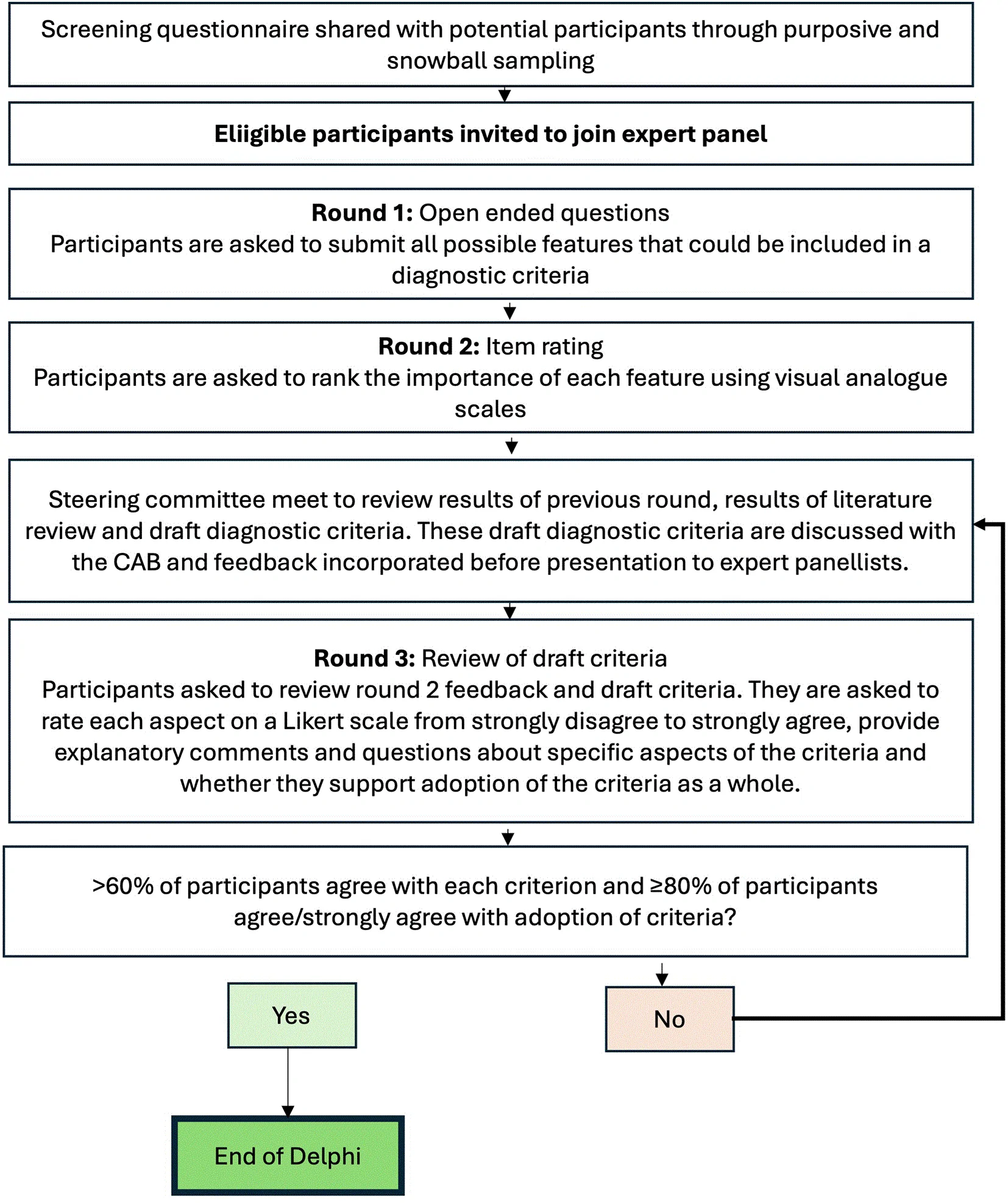
Flow of Delphi Rounds and criteria for termination of study.

The flow of Delphi rounds is shown in Figure 2. Following their invitation, panellists will be invited to round 1. At the start of each round, panellists will view be asked to re-review the participant information sheet and be asked to confirm their consent before completing the online survey.

**Round 1: Initial item generation**

At the start of round 1 they will be asked to watch a video explainer of the study before proceeding to the survey. Panellists will be asked to contribute all possible features that they think could be relevant to include in the diagnostic criteria. They will be asked open and closed questions about diagnosis of aEV in their practice.

At the end of each survey the participant will be asked to confirm their email address, this allows their responses to be linked to those of previous surveys and the research team to monitor attrition.

**Round 2: Item rating**

Panellists will be asked to rate the importance of each possible feature using a visual analogue scale from “not at all important” to “essential”. They will also be asked to rate each features sensitivity for aEV “always present” to “rarely present”, and specificity “if present diagnosis can only be EV” to “often present in skin conditions that are not aEV”. Panellists will be given space to provide comments about the features, and the possible groupings of features that may be included in the draft diagnostic criteria.

Features with a median value higher than 60/100 will be considered for inclusion in the draft diagnostic criteria by the steering committee. Those with scores between 60 and 79 will be included in the draft “supportive” criteria; to reflect that they are present in some but not all cases of aEV. In line with similar studies, all features with a median of ≥80/100 will be included in the draft diagnostic criteria. ^24^ The steering committee will discuss each feature for inclusion in either the “essential” or “supportive“ criteria. The final decision will rest with the steering committee to reflect that some features may be consistent but still may not be essential to diagnosis. ^24^

The steering committee will also review a collated summary of the published literature following round 2. The features present in the literature review will be compared to those rated as important by the panel. If discrepancies are present, these will be discussed and features added for rating by the expert panellists in round 3 if necessary. A record of these discussions will be maintained for transparency.

**Round 3: Review of draft criteria**

Panellists will be presented with de-identified collated feedback from round 2, draft screening and diagnostic criteria. They will be asked to rate their level of agreement with each feature of the diagnostic criteria in terms of relevance on a 5-point Likert scale and their overall level of agreement with the diagnostic criteria. Panellists will be asked to provide free-text comments to explain any disagreements, suggested changes to phrasing or terminology. Panellists will be given the option to not respond if they feel an area is outside of their expertise.

The steering committee will meet to review these results prior to round 4. Criteria that do not meet moderate consensus (<60% agreement) will not be taken forward to the next round. Those that reach moderate consensus (≥60-79%) consensus will be refined prior to the next round. Those that reach strong consensus (≥80%) will be included in the next round and will be refined based on the free text comments.

**Round 4: Further refinement of draft criteria**

Panellists will be presented feedback from round 3 and a refined diagnostic criteria based on the feedback from round 3. They will be asked to rate their agreement with the changes and their agreement with adoption of the overall criteria.

### Definition of consensus and non-consensus

The definition of moderate consensus is 60–79% and strong consensus is ≥80% agreement with agreement defined as the percentage of positive responses (agree or strongly agree) compared to the total completed responses. ^24^ The study will be terminated once at least moderate consensus is met for each feature of the diagnostic criteria **and** strong consensus for the adoption of the overall criteria is met.

### Handling of non-consensus items

In round 2 panellists will be asked to rank possible diagnostic features on a visual analogue scale (0–100) as described above. Median values will be calculated for each feature. All features with a median value ≥80 will be included in the draft diagnostic criteria. Features with a median less than 60 will not be included in the draft criteria. This ensures that features not considered of at least moderate importance are not included in the draft criteria and is aligned with similar studies in the field. ^24^

All features whose median value falls between 60 and 79 will be retained and discussed for inclusion by the steering committee in the context of any free-text comments and review of the published literature. The steering committee will decide on whether to include them in the draft criteria.

In round 3, panellists will rate their agreement with the structure and content of the draft criteria using Likert-style questions. Items that do not meet the moderate consensus threshold i.e. less than 60% of participants either agree or strongly agree will not progress to the next round a priori. If free text comments and steering committee suggest that they should be included with significant revision, the revised versions may be included in the next round. The panellists will be asked to provide comments where they disagree with any criteria and suggestions regarding structure, syntax, clarity of phrasing and terminology. These will be used to guide the revisions prior to the next round.

The steering committee will revise the draft criteria based on analysis of the results from round 3 and the free text comments. The revised criteria are presented to the panel in the next round.

### Handling of non-response

Participants who have not responded to the invitation for round 1, will be sent a further reminder at 3 days, 1 week, 10 days and 14 days. Those who have not responded to the invitation at 17 days will be considered a non-responder for that round.

Panellists will be sent an email reminder one week before the start of round 2 and all subsequent rounds, containing the collated feedback of the previous round. With the exception of round 1, all rounds will be open for 2 weeks. Participants will be sent a reminder email one week, two days and one day before closure. Those who have opted in to phone reminders will be sent a WhatsApp message by the study facilitator reminding them complete the survey. WhatsApp is widely used to communicate between healthcare professionals globally as an adjunct to email communication. ^31– 33^ It is not country specific and is end-to-end encrypted. The use of WhatsApp in addition to email as means of professional communication is equitable and is likely to increase participation. ^32^ Those who have not responded at the time of round closure will be sent an email giving them a further three days to complete the survey. If they do not respond by three days they will be considered a non-responder for that round.

The proportion of responders in each round will be reported; a minimum acceptable response rate will be set at 70% or a minimum of 15 participants for each round. If this threshold is not met the round deadline will be extended. Non-responders for one round will not be excluded from the study and will continue to be invited to respond in ongoing rounds of the study.

### Handling of disagreement between the panel responses and CAB input

CAB input will be sought at key points throughout the study. The CAB will be asked for their feedback on the draft diagnostic criteria following round 3. If there is significant disagreement between the CAB members responses and the responses of the panellists, this will be discussed by the steering committee. The steering committee will retain the final decision. If there is significant deviation, then this will be recorded with written justification and fed back to the CAB who will be given opportunity to respond.

### Criteria for termination

The Delphi surveys will be carried out iteratively until strong consensus (>80%) is reached on the adoption of the draft criteria as a whole and moderate consensus is met for each individual criterion. ^21, 34^ If there is stable non-consensus after 5 rounds with stability defined as an interquartile range of responses between rounds <10%, the steering committee will meet and consider a synchronous teleconference with the Delphi panellists to seek resolution on the key issues.

### Preliminary validation exercise

Once consensus on the diagnostic criteria has been reached, two members of the steering committee will independently go through each biopsy confirmed case identified in the literature review and apply the agreed criteria based on reported clinical findings and/or photographs. The proportion meeting the consensus criteria will be reported and any cases not meeting the criteria discussed and reported in detail.

## Data analysis

### Analysis of quantitative data

In round 1 all responses from panellists will be collated and presented to panellists in round 2. In round 2, median and interquartile range will be calculated for the score of each feature. In round 3 we will present the proportion of panellists reporting each response, the median response and the interquartile range.

### Analysis of qualitative data

Qualitative data will be generated in the free text response in round 1 and in the free text options in rounds 2 onwards. The free text outputs will be coded by the steering committee, and a thematic analysis will be carried out. Any themes identified will be presented to the steering committee and will be considered in the revision of the draft criteria for future rounds.

The study team will analyse data based on study number unlinked to demographic information, to minimise the impact of authority bias.

## Data management

All data will be collected and managed using REDCap hosted at London School of Hygiene and Tropical Medicine. ^29^ Participants will be assigned a study ID on the first questionnaire linked to their email address. Two data sets will be exported from REDCap. A de-identified data set containing participant ID and survey responses. A pseudo anonymisation key containing participant ID, name and email address. This data will be kept on a password protected file on LSHTM secure server.

At the end of the study the pseudo anonymisation key will be deleted. Participants will be given the option to opt in to ongoing communication about ongoing manuscripts and to have their names acknowledged in any published manuscript. Those that opt into this will have their information retained in a new excel file on the secure server until publication of the study. Participants who do not opt into this will not have their name or contact details retained.

The de-identified dataset will be stored on the secure server for 10 years after study completion in line with Wellcome policy.

## Ethical issues

The study has been approved by LSHTM research ethics committee (Reference: 33189). The main ethical issues are summarised below.

• **Data protection:** Data will be stored securely on an LSHTM server in line with GDPR and university data protection policy. Confidential data will only be accessible to the study team. All panellists will give written informed consent prior to participation in round one and will be asked to confirm this before each round.
• **Confidentiality:** Panellists’ responses will not be identifiable to other panellists during the Delphi rounds. This will be achieved by aggregating the feedback from each round and summarising the overall comments. No comments will be assigned to an individual. Panellists will not be told who the other contributors to the Delphi study are. In recognition of the work and expertise that is required to contribute to the Delphi study, all panellists will be given the option to have their contribution acknowledged in the final published paper.
• **Structural barriers:** The structural barriers to participation will be minimised by the virtual format and by using REDCap which allows participants to save and return to the survey and by giving reminders on WhatsApp as well as email in line with participant preference.
• **Informed consent:** The informed consent process is explained above. Electronic consent forms will be used to enable participation of experts globally. This will be facilitated using REDCap signatures and eConsent feature. ^30^
• **Conflict of interest:** There are no known conflicts of interest. All panellists will be asked to declare any conflicts of interest and to recluse themselves if there is a significant conflict of interest.

### Public involvement

Community involvement has been sought from an independent study community advisory board (CAB) – this CAB is a group of adults living with HIV and skin disease. These colleagues are well placed to advise on the acceptability and usability of any proposed diagnostic criteria and will be involved in finalising the questionnaires and dissemination of results.

### Dissemination plan

The final agreed diagnostic criteria will be shared through publication in journals, presentation at international dermatology and HIV conferences and dissemination through dermatology networks such as GloDerm, Regional Dermatology Training Centre (Tanzania) and International League of Dermatology Societies. We plan to evaluate the diagnostic criteria for accuracy and real-world utility by applying them to biopsy proven cases reported in the literature and utilising them in a skin survey as part of a separate study.

## Discussion

HIVE MIND is the first study to attempt to formalise diagnosis of aEV using expert consensus. This aligns clearly with the WHA resolution to prioritise research into skin disease as a global health priority. ^19^ In particular, this study comes as a response to the lack of prevalence data at an international and national level and the diversity of definitions for aEV in the literature. ^1, 8, 12, 35^

HIVE MIND also speaks to the growing recognition of the health needs of young people living with HIV beyond viral suppression. ^36, 37^ Skin disease is visible and stigmatising for young people with HIV, but despite its prevalence is rarely addressed. ^6, 16^ A diagnosis can be useful for the individual and community. Allowing the community to better understand their condition and to advocate for research and treatment. ^38^ It is also useful on a clinic and national level, allowing policy makers to better understand the needs of their population. At present the risk of progression to malignancy in patients with aEV is not known and cannot be assumed to be the same as that of those with congenital EV. ^8, 16^ An agreed definition of aEV is the first step in designing epidemiological studies to assess this.

HIVE MIND is specifically designed to enable diagnosis across a range of settings and to recognise that the diagnostic tests possible in the context of research are often not feasible where the burden of disease is the highest. This design ensures that the diagnostic criteria developed are useful to the clinicians and communities with the most need. Widespread applicability is of even more importance for rare conditions where standardisation allows comparison of clinical outcomes across borders. ^24^

Whilst HIVE MIND has several strengths, there are limitations. Delphi methodology by its nature relies on expert opinion and is therefore highly dependent on the expertise of the panel and their willingness to engage in the Delphi process. The sample size is intentionally low, in order to allow recruitment of engaged participants with true expertise in this area. The diagnostic criteria developed will need to be validated in future work before being widely adopted.

In summary, HIVE MIND is the essential first step in increasing recognition and research into a neglected condition. It is the first study to bring together a pool of global experts to share their expertise on aEV and provides the essential first step in future research to estimate the prevalence of aEV, understand the malignancy risk and best treatments.

## Data Availability

This is a protocol and as such there are no underlying data associated with this manuscript. The extended data associated with this article are available on LSHTM Data Compass. LSHTM Data Compass:Extended data for HIVE MIND - Establishing globally relevant diagnostic criteria for HIV associated acquired epidermodysplasia verruciformis: an international Delphi study. https://doi.org/10.17037/data.00005155. ^28^ This project contains the following extended data in an appendices file:
• A list of variables extracted in the literature review • Participant information sheets and consent forms • Questionnaire for the first survey round Data are available under the terms of the Creative Commons Zero “No rights reserved” data waiver (CC0 1.0 Public domain dedication). The study has been designed and will be reported in accordance with the ACcurate COnsensus Reporting Document (ACCORD), Appendix 2. ^23^
