# Supplementary material for: HIVE MIND - Establishing diagnostic criteria for HIV associated acquired epidermodysplasia verruciformis: an international Delphi study
Source: Wellcome Open Res. 2026 Aug 29;11:276. Originally published 2026 May 11. [Version 3] doi: 10.12688/wellcomeopenres.26218.3 (PMC13555086; doi:10.12688/wellcomeopenres.26218.3)
Supplement: Peer review history

# Reviewer report: Reviewer response for version 2

**DOI:** 10.21956/wellcomeopenres.29831.r160213

**Published:** 2026-07-22

**Authors:** Luis Alberto Ribeiro Fróes Júnior

**Affiliations:** 1 Universidade de Sao Paulo, São Paulo, State of São Paulo, Brazil

This is a relevant and well-motivated protocol addressing an important diagnostic gap. The modified Delphi design is appropriate, but two methodological issues require clarification.

 First, individual diagnostic criteria may be retained with only 60% agreement. This threshold permits substantial disagreement within the panel. The authors should provide a stronger justification or require strong consensus for core diagnostic criteria, with moderate-consensus items classified separately as supportive features.

 Second, the steering committee retains considerable discretion in coding and consolidating Round 1 responses, integrating the literature review, and deciding whether to include items scoring 60–79. These procedures should be predefined and reported transparently. The protocol should also include a formal comparison between panel-generated items and the literature review to identify potentially omitted features.

 The claim of global relevance should be moderated because representation outside the African region may consist of only one participant per WHO region. External validation will remain necessary before the criteria can be considered broadly generalizable.

Is the study design appropriate for the research question?

Yes

Is the rationale for, and objectives of, the study clearly described?

Yes

Are sufficient details of the methods provided to allow replication by others?

Partly

Are the datasets clearly presented in a useable and accessible format?

Not applicable

Reviewer Expertise:

Dermatology, tropical and infectious dermatology,

I confirm that I have read this submission and believe that I have an appropriate level of expertise to confirm that it is of an acceptable scientific standard, however I have significant reservations, as outlined above.

# Response

**Published:** 2026-08-05

**Authors:** Sophie Kelly

**Affiliations:** Non-communicable Diseases Epidemiology, London School of Hygiene and Tropical Medicine Faculty of Epidemiology and Population Health, London, England, UK

*We would like to thank the reviewer for taking the time to thoroughly review our protocol. We hope that we have adequately addressed the concerns raised with the changes set out below.* This is a relevant and well-motivated protocol addressing an important diagnostic gap. The modified Delphi design is appropriate, but two methodological issues require clarification.

**Comment 1**

 First, individual diagnostic criteria may be retained with only 60% agreement. This threshold permits substantial disagreement within the panel. The authors should provide a stronger justification or require strong consensus for core diagnostic criteria, with moderate-consensus items classified separately as supportive features.

*We have clarified this. The “Essential” criteria require over 80% agreement, suggesting that they are nearly always present in cases of AEV; “Supportive” criteria will require over 60% agreement allowing features to be included that are supportive but not present in all cases. *   “Features with a median value higher than 60/100 will be considered for inclusion in the draft diagnostic criteria by the steering committee. Those with scores between 60 and 79 will be included in the draft “supportive” criteria; to reflect that they are present in some but not all cases of aEV. In line with similar studies, all features with a median of ≥80/100 will be included in the draft diagnostic criteria. ^24  ^The steering committee will discuss each feature for inclusion in either the “essential” or “supportive” criteria. The final decision will rest with the steering committee to reflect that some features may be consistent but still may not be essential to diagnosis.”

**Comment 2**

 Second, the steering committee retains considerable discretion in coding and consolidating Round 1 responses, integrating the literature review, and deciding whether to include items scoring 60–79. These procedures should be predefined and reported transparently. The protocol should also include a formal comparison between panel-generated items and the literature review to identify potentially omitted features.     “The steering committee will also review a collated summary of the published literature following round 2. The features present in the literature review will be compared to those rated as important by the panel. If discrepancies are present, these will be discussed and features added for rating by the expert panellists in round 3 if necessary. A record of these discussions will be maintained for transparency.”

*We have added a step at the end of the process where the case report literature is reviewed to see the proportion biopsy confirmed cases would meet the criteria based on their reported findings and photographs included.   *   “Preliminary Validation Exercise Once consensus on the diagnostic criteria has been reached, two members of the steering committee will independently go through each biopsy confirmed case identified in the literature review and apply the agreed criteria based on reported clinical findings and/or photographs. The proportion meeting the consensus criteria will be reported and any cases not meeting the criteria discussed in detail.”

**Comment 3**

 The claim of global relevance should be moderated because representation outside the African region may consist of only one participant per WHO region. External validation will remain necessary before the criteria can be considered broadly generalizable.

*We have moderated this claim in the title and throughout the text. We have added a preliminary validation step based on the published literature but have plans for external validation following this process in a separate study.*

*The title now reads:* *HIVE MIND - Establishing diagnostic criteria for HIV associated acquired epidermodysplasia verruciformis: an international Delphi study.*    

 Many thanks for your input.

   Yours sincerely, Sophie Kelly on behalf of all co-authors

# Reviewer report: Reviewer response for version 2

**DOI:** 10.21956/wellcomeopenres.29831.r159617

**Published:** 2026-07-17

**Authors:** Tarun Narang

**Affiliations:** 1 Post Graduate Institute of Medical Education and Research, Chandigarh, Punjab, India

I thank the authors for their thorough response and for addressing the suggestions in the revision cycle. The modifications made to the manuscript have successfully resolved the queries raised in the previous review, enhancing both the clarity and the strength of the paper. I am satisfied with the revisions.

Is the study design appropriate for the research question?

Yes

Is the rationale for, and objectives of, the study clearly described?

Yes

Are sufficient details of the methods provided to allow replication by others?

Yes

Are the datasets clearly presented in a useable and accessible format?

Not applicable

Reviewer Expertise:

Dermatology , Tropical skin infections

I confirm that I have read this submission and believe that I have an appropriate level of expertise to confirm that it is of an acceptable scientific standard.

# Reviewer report: Reviewer response for version 2

**DOI:** 10.21956/wellcomeopenres.29831.r159618

**Published:** 2026-07-13

**Authors:** I Made Dwi Mertha Adnyana

**Affiliations:** 1 Universitas Jambi, Jambi, Indonesia

1. This event is to be attended by at least 15 participants from Africa, as well as at least one participant from each of the other four regions. It is clear that one participant per region is a very low threshold for claiming meaningful regional clinical representation. The authors must tone down the claim of "globally relevant" in the title to a more precise phrase, such as "criteria informed by a globally representative but Africa-weighted panel."

 2. The additional sentences merely cite two systematic reviews without synthesising their main findings (lesion types, geographic distribution of cases, and reported diagnostic limitations). Add two to three sentences summarising the findings of Cuestas et al. (2024) and Moore et al. (2022) to strengthen the empirical basis of the introduction.

 3. The author's response in the reply letter was scientifically honest regarding the risk of malignancy associated with aEV versus classic EV, but that statement does not appear to have been directly included in the "Discussion" or "Limitations" sections of the manuscript. An explicit sentence must be added to the "Limitations" section stating that the risk of malignancy associated with aEV cannot yet be assumed to be equivalent to that of congenital EV.

 4. The argument for avoiding priming bias is consistent with the principles of the classical Delphi method (Jünger et al., CREDES 2017). The protocol does not yet explain the mechanism for the completeness check. Specifically, it does not say whether the steering committee will compare the list of items from Round 1 against the findings of the literature review. This would be used to identify aEV phenotypes that may have been overlooked by the panel, prior to drafting Round 3. It is imperative that this step is added to verify the completeness of the literature case data.

 5. The CAB's role has been explained, but the resolution mechanism for cases where the CAB's input differs from the expert panel's rating has not yet been outlined in procedural terms. We recommend adding a specific escalation process. The steering committee must retain the final decision, and written justification must be provided if it deviates from the CAB's input.

Is the study design appropriate for the research question?

Yes

Is the rationale for, and objectives of, the study clearly described?

Partly

Are sufficient details of the methods provided to allow replication by others?

Partly

Are the datasets clearly presented in a useable and accessible format?

Partly

Reviewer Expertise:

Tropical Medicine

I confirm that I have read this submission and believe that I have an appropriate level of expertise to confirm that it is of an acceptable scientific standard, however I have significant reservations, as outlined above.

# Response

**Published:** 2026-08-05

**Authors:** Sophie Kelly

**Affiliations:** Non-communicable Diseases Epidemiology, London School of Hygiene and Tropical Medicine Faculty of Epidemiology and Population Health, London, England, UK

Dear Reviewer, We *would like to thank you for your ongoing review. We have made significant improvements based on the comments provided and believe that the concerns raised have been adequately addressed. *  

**Comment 1 ** This event is to be attended by at least 15 participants from Africa, as well as at least one participant from each of the other four regions. It is clear that one participant per region is a very low threshold for claiming meaningful regional clinical representation. The authors must tone down the claim of "globally relevant" in the title to a more precise phrase, such as "criteria informed by a globally representative but Africa-weighted panel."  

*We have moderated this claim in both the title and subsequent text.  The title now reads* *: HIVE MIND - Establishing diagnostic criteria for HIV associated acquired epidermodysplasia verruciformis: an international Delphi study. *  

**Comment 2**

 The additional sentences merely cite two systematic reviews without synthesising their main findings (lesion types, geographic distribution of cases, and reported diagnostic limitations). Add two to three sentences summarising the findings of Cuestas et al. (2024) and Moore et al. (2022) to strengthen the empirical basis of the introduction.  

*We have made some changes to the structure of the introduction to improve flow and added the following text: *   “Clinical and scientific knowledge of HIV-associated aEV is predominantly based on descriptions of patients in case report and case series, small cohort studies and two systematic reviews. ^4,5^ Sixty percent (n=48) of the 80 reported cases in the systematic review by Cuestas et al., were in people from Africa. ^4^ The remainder of the cases were from the Americas (n = 17), Europe (n=14) and Asia (n=1). ^4^ The described clinical presentation was predominantly of widespread flat warts, hypopigmented macules and pityriasis versicolour-like lesions across the face upper limbs and upper trunk. ^4,5^ There was no standard method of diagnosis, with biopsy carried out in 37.5% of cases and HPV typing carried out in 73.5%; the most detected genotypes were 5, 20 and 8. ^4^”  

**Comment 3** The author's response in the reply letter was scientifically honest regarding the risk of malignancy associated with aEV versus classic EV, but that statement does not appear to have been directly included in the "Discussion" or "Limitations" sections of the manuscript. An explicit sentence must be added to the "Limitations" section stating that the risk of malignancy associated with aEV cannot yet be assumed to be equivalent to that of congenital EV.  

*Many thanks for this comment. We have added the following sentences to the introduction and discussion to make this clear. *  

**Introduction ** “This is not routine practice for patients with HIV-associated aEV in the global south and, although some cases have been reported, the rate of progression to malignancy is not known. ^4,15–18”^  

**Discussion** “At present, the risk of progression to malignancy in patients with aEV is not known and cannot be assumed to be the same as that of those with congenital EV. An agreed definition of aEV is the first step in designing epidemiological studies to assess this.”

**Comment 4**

 The argument for avoiding priming bias is consistent with the principles of the classical Delphi method (Jünger et al., CREDES 2017). The protocol does not yet explain the mechanism for the completeness check. Specifically, it does not say whether the steering committee will compare the list of items from Round 1 against the findings of the literature review. This would be used to identify aEV phenotypes that may have been overlooked by the panel, prior to drafting Round 3. It is imperative that this step is added to verify the completeness of the literature case data.  

*Many thanks. We have added the following paragraph to make this clearer.  *   “The steering committee will also review a collated summary of the published literature following round 2.

 The features present in the literature review will be compared to those rated as important by the panel. If discrepancies are present, these will be discussed and features added for rating by the expert panellists in round 3 if necessary.”  

*We have also added a preliminary validation step: * “Preliminary Validation Exercise Once consensus on the diagnostic criteria has been reached, two members of the steering committee will independently go through each biopsy confirmed case identified in the literature review and apply the agreed criteria based on reported clinical findings and/or photographs. The proportion meeting the consensus criteria will be reported and any cases not meeting the criteria discussed and reported in detail.”    

**Comment 5 ** The CAB's role has been explained, but the resolution mechanism for cases where the CAB's input differs from the expert panel's rating has not yet been outlined in procedural terms. We recommend adding a specific escalation process. The steering committee must retain the final decision, and written justification must be provided if it deviates from the CAB's input.

*We have added this step to the methods - many thanks. *   “CAB input will be sought at key points throughout the study.  The CAB will be asked for their feedback on the draft diagnostic criteria following round 3.  If there is significant disagreement between the CAB members responses and the responses of the panellists, this will be discussed by the steering committee. The steering committee will retain the final decision. If there is significant deviation, then this will be recorded with written justification and fed back to the CAB who will be given opportunity to respond.”    

 Many thanks again for your input. 

 Yours sincerely, Sophie Kelly on behalf of the co-authors

# Reviewer report: Reviewer response for version 1

**DOI:** 10.21956/wellcomeopenres.28878.r155500

**Published:** 2026-06-02

**Authors:** I Made Dwi Mertha Adnyana

**Affiliations:** 1 Universitas Jambi, Jambi, Indonesia

Dear Authors, 

 The reason for this study is clear, and there is a need for this research. The way the protocol is set out makes sense. However, there are some problems with the study that need to be fixed before it can continue. These problems include the fact that the results are not consistent, there is no minimum number of people who have to take part, there is not enough information about the size of the sample, there is no explanation about why the study is being done, and there is no way to check the results.

**Major Concern**

 1. The title says that the criteria are "globally relevant", but the recruitment strategy does not guarantee the minimum number of people from each WHO region. The phrase "globally relevant" is not justified by the design unless the number of items per region is clearly set and enforced, not just as a goal.

 2. There is no accepted way to diagnose aEV, and because it is not often diagnosed, researchers have been unable to study how common it is, how it is treated, and whether it can become cancerous. However, the introduction does not give a step-by-step summary of how existing case series or reviews have worked out what aEV is.

 3. The phrase "increased vulnerability to cutaneous human papillomaviruses" should be explained in more detail. Does this show that the immune system is weak against certain types of HPV, or does it show a general weakening of the immune system? This will directly affect which tests are possible.

 4. The authors do not explain if the risk of cancer from aEV is expected to be different from that of classic EV, given that they are caused by different things (T-cell immunosuppression from HIV versus germline EVER1/TMC6 or EVER2/TMC8 mutations). This difference has a big impact on how important it is to set up rules for monitoring.

 5. The authors say that they use Delphi methodology because aEV is rare and there is not much data to go on. This is a good reason, but it is not enough. The rules do not say if a formal review was done to find out what is already known about the topic before the Delphi. If such a review was done, the results should be used to create the first list of items. If you don't do this, it makes the reasons for the item generation approach in Round 1 weaker.

 6. The rules say that consensus a priori is when more than 60% of people agree on each individual thing, and when more than 80% of people agree on everything together. But the section called 'Criteria for Termination' says that we keep going until everyone agrees on the draft rules (more than 80% of people). It doesn't say if this is for each thing or for all of them together. If 63% of people agree, it is much weaker than if 85% agree. But the current language says that both of these would end the study. The logic at the start must be made clear so that we can decide if items that only get a moderate agreement (60–79%) need more review, separate from the overall vote on adoption.

 7. The rules say that people on the panel must "add all the features they think are important". If we don't have a list of seeds that's based on a review of what's already been published, Round 1 might produce a set of items that are all very different from each other and show what the panellists have seen in their own practice, rather than covering all the different types of aEV phenotypes that have been published. This is particularly risky because the panel is small and the clinical experience is not spread evenly across different places. The authors should explain if the review of other studies' findings will be given to the people on the panel before or with Round 1, and if any fixed-format item domains will create a structure for the open-ended answers.

 8. The study's design has been discussed with a community advisory board made up of people with personal experience of skin disease and HIV. The CAB's role is to provide advice at specific points in the process, from the design stage to when the results are shared. However, the protocol does not clearly explain when the CAB's input is officially requested or how disagreements between CAB feedback and expert panel ratings will be resolved. If we don't have this step-by-step plan, CAB involvement might just be a temporary fix instead of being a long-term part of the structure.

 9. The protocol creates a set of rules that everyone agrees on, but it does not include checking these rules against cases that have been confirmed by a biopsy as part of the study. The dissemination section mentions future application to cases where a biopsy has been done, but this is presented as a separate study. When we are trying to come up with rules that can be used in places where there is not much information, and we cannot do a biopsy, it is very important to be aware of the fact that we do not have any plans to estimate how good these rules are. We should talk about this in the Discussion.

**Minor concern**

 1. CREDES compliance: The protocol says that you must follow the CREDES guidance (that's Conducting and Reporting of Delphi Studies, by the way), but it doesn't show exactly how each part of the protocol fits with the CREDES checklist items. A supplementary compliance table would make the methods used clearer.

 2. Ethics section: The ethical approval reference (LSHTM REC 33189) is provided, but the protocol does not address compensation or data privacy for participants from outside the UK, which is relevant given the recruitment strategy across multiple regions, including sub-Saharan Africa.

 3. WhatsApp reminders: If you have agreed to receive a phone reminder, the study facilitator will send you a WhatsApp message to remind you. If you want to use a commercial, non-GDPR-compliant messaging platform to contact the participants, you need to have a good reason and get the go-ahead from the ethics committee, which is not mentioned.

Is the study design appropriate for the research question?

Yes

Is the rationale for, and objectives of, the study clearly described?

Partly

Are sufficient details of the methods provided to allow replication by others?

Partly

Are the datasets clearly presented in a useable and accessible format?

Partly

Reviewer Expertise:

Tropical Medicine

I confirm that I have read this submission and believe that I have an appropriate level of expertise to state that I do not consider it to be of an acceptable scientific standard, for reasons outlined above.

# Response

**Published:** 2026-06-18

**Authors:** Sophie Kelly

**Affiliations:** Non-communicable Diseases Epidemiology, London School of Hygiene and Tropical Medicine Faculty of Epidemiology and Population Health, London, England, UK

*Dear Reviewer,* *We would like to thank the reviewer for taking the time to complete a thorough review. We have used this review to make significant improvements to the manuscript  (uploaded as version 2). We hope that these changes adequately address your concerns.*

**Major Concern**

 1. The title says that the criteria are "globally relevant", but the recruitment strategy does not guarantee the minimum number of people from each WHO region. The phrase "globally relevant" is not justified by the design unless the number of items per region is clearly set and enforced, not just as a goal.

*We thank the reviewer for their comment. We have adjusted our recruitment strategy to include at least one participant from four WHO regions and at least 15 participants from the African WHO region. We have purposively selected for an over-representation of participants from the African region to reflect the likely distribution of cases of HIV-associated aEV and where there is most need for clinical diagnostic criteria. We appreciate that this may mean our criteria our less suitable for the other WHO regions, given the paucity of cases reported in these regions and the greater availability we believe that this is acceptable.  * Line 167 “To reflect the need for internationally applicable diagnostic criteria, recruitment will focus on members from all WHO regions; with a particular drive to increase participation from the WHO Africa Region as this region has a disproportionate burden of HIV. We will therefore only close recruitment when at least 15 participants from the WHO African region have joined the panel.”

 2. There is no accepted way to diagnose aEV, and because it is not often diagnosed, researchers have been unable to study how common it is, how it is treated, and whether it can become cancerous. However, the introduction does not give a step-by-step summary of how existing case series or reviews have worked out what aEV is.

*Many thanks, we have updated the introduction to make this clear. * Line 8: “Clinical and scientific knowledge of aEV is predominantly based on descriptions of patients in case report and case series, small cohort studies and two recent systematic reviews. ^4,5^”

 3. The phrase "increased vulnerability to cutaneous human papillomaviruses" should be explained in more detail. Does this show that the immune system is weak against certain types of HPV, or does it show a general weakening of the immune system? This will directly affect which tests are possible.

*Many thanks for this comment. The mechanisms by which immunosuppression in the context of HIV contributes to aEV is not fully elucidated. There is some work showing that late HIV diagnosis leads to incomplete immune reconstitution in the skin compartment (Saluzzo et al.,), but this does not full explain why this phenomenon is observed more commonly in people with perinatally acquired HIV. We expect this is due to a depleted CD4 count at the time of beta HPV exposure, shortly after birth. The introduction has been expanded to reflect this. * Line 24: “The exact mechanism of development of aEV in people with HIV is not fully elucidated. There is evidence that immune reconstitution in the skin compartment is not complete in those who are diagnosed with a CD4 ≤350 cells/mm3 ^4^. Whilst this alone does not explain the disproportionate number of cases observed in people with perinatally acquired HIV, a hypothesis is that a poor T cell response upon first exposure to beta-HPVs as an infant or child increases the risk of beta-HPV persistence and aEV development ^4,10,11^.

 4. The authors do not explain if the risk of cancer from aEV is expected to be different from that of classic EV, given that they are caused by different things (T-cell immunosuppression from HIV versus germline EVER1/TMC6 or EVER2/TMC8 mutations). This difference has a big impact on how important it is to set up rules for monitoring.

*It is not known at present whether the risk of malignancy is expected to be different from classic EV and there have been no epidemiological studies to explore this. The aim of this Delphi is not to set up rules for treatment monitoring but to agree consensus diagnostic criteria. This is an essential first step prior to larger scale epidemiological studies.*

 5. The authors say that they use Delphi methodology because aEV is rare and there is not much data to go on. This is a good reason, but it is not enough. The rules do not say if a formal review was done to find out what is already known about the topic before the Delphi. If such a review was done, the results should be used to create the first list of items. If you don't do this, it makes the reasons for the item generation approach in Round 1 weaker.

*Many thanks for your comment. We discussed this carefully in planning this study. We have purposefully not used this to influence our panel prior to round 1 – initial item generation. The published body of evidence has systemic biases in that is based on case reports, predominantly from higher income and lower HIV incidence settings (USA, Spain and France) and two paediatric cohort studies from Zimbabwe and Botswana that may not be representative of the wider picture. Priming the panel with the features mentioned in this relatively small, biased sample may unduly influence their responses to round 1. We explicitly want them to contribute features of importance based on their clinical experience.* *We do however plan to use the current literature to feed into the draft diagnostic criteria. Please see Line 213 in which we detail how we will carry out a literature review and use this to inform the development of draft criteria after round 2.*   Line 240: The literature review will not be circulated to the participants prior to round 1 to avoid influencing their opinions. The majority of cases reported in the literature come from case reports in high resource settings, where presentation and access to tests may be different from the experience of our panellists. The diagnostic criteria developed using this Delphi are intended to be based on the clinical experience of clinicians who see this condition regularly and not unduly influenced by cases presented in case reports. 

 6. The rules say that consensus a priori is when more than 60% of people agree on each individual thing, and when more than 80% of people agree on everything together. But the section called 'Criteria for Termination' says that we keep going until everyone agrees on the draft rules (more than 80% of people). It doesn't say if this is for each thing or for all of them together. If 63% of people agree, it is much weaker than if 85% agree. But the current language says that both of these would end the study. The logic at the start must be made clear so that we can decide if items that only get a moderate agreement (60–79%) need more review, separate from the overall vote on adoption.

*Many thanks, we have made this clearer and updated the manuscript.* Line 311 The steering committee will meet to review these results prior to round 4. Criteria that do not meet moderate consensus (<60% agreement) will not be taken forward to the next round. Those that reach moderate consensus  (≥60-79%) consensus will be refined prior to the next round. Those that reach strong consensus (≥80%) will be included in the next round and will be refined based on the free text comments.   Line 339 In round 3, panellists will rate their agreement with the structure and content of the draft criteria using Likert-style questions. Items that do not meet the moderate consensus threshold i.e. less than 60% of participants either agree or strongly agree will not progress to the next round a priori. If free text comments and steering committee suggest that they should be included with significant revision, the revised versions may be included in the next round.

 7. The rules say that people on the panel must "add all the features they think are important". If we don't have a list of seeds that's based on a review of what's already been published, Round 1 might produce a set of items that are all very different from each other and show what the panellists have seen in their own practice, rather than covering all the different types of aEV phenotypes that have been published. This is particularly risky because the panel is small and the clinical experience is not spread evenly across different places. The authors should explain if the review of other studies' findings will be given to the people on the panel before or with Round 1, and if any fixed-format item domains will create a structure for the open-ended answers.

*We purposively recruit a highly experienced panel and do not want to influence their decisions based on the published literature. The published literature is inherently biased and disproportionately reflects cases in the global north and those with atypical presentations. The aim of this exercise is to use real world experience of clinicians who see this condition regularly to inform diagnostic criteria. We are clear about how we will use a review of the literature to inform the first draft diagnostic criteria following round 2.*

 8. The study's design has been discussed with a community advisory board made up of people with personal experience of skin disease and HIV. The CAB's role is to provide advice at specific points in the process, from the design stage to when the results are shared. However, the protocol does not clearly explain when the CAB's input is officially requested or how disagreements between CAB feedback and expert panel ratings will be resolved. If we don't have this step-by-step plan, CAB involvement might just be a temporary fix instead of being a long-term part of the structure.

*Many thanks. We have updated the protocol to make the role of the CAB clearer. They will be involved throughout the study with key review points. We have updated this in line 137:*  “They will be consulted at all stages of the study and have been involved in design of the protocol. The study facilitator will liaise with the CAB at key points throughout the study (including design phase, to present draft diagnostic criteria, after expert agreement of criteria, dissemination phase) to ensure inclusivity and that the final diagnostic criteria are acceptable to people living with aEV.  If necessary, the CAB will be invited to present their discussions to the steering committee and vice versa.”

 9. The protocol creates a set of rules that everyone agrees on, but it does not include checking these rules against cases that have been confirmed by a biopsy as part of the study. The dissemination section mentions future application to cases where a biopsy has been done, but this is presented as a separate study. When we are trying to come up with rules that can be used in places where there is not much information, and we cannot do a biopsy, it is very important to be aware of the fact that we do not have any plans to estimate how good these rules are. We should talk about this in the Discussion.

*Many thanks for this comment. The aim of this study is to develop consensus on diagnostic criteria for aEV. These criteria will be validated in future work, but this is outside of the scope of this project. This has been acknowledged further in the discussion. * Line 482 “The diagnostic criteria developed will need to be validated in future work before being widely adopted.”

**Minor concern**

 1. CREDES compliance: The protocol says that you must follow the CREDES guidance (that's Conducting and Reporting of Delphi Studies, by the way), but it doesn't show exactly how each part of the protocol fits with the CREDES checklist items. A supplementary compliance table would make the methods used clearer.

*Many thanks for spotting this; following discussion with co-authors, it has been decided that the ACCORD checklist is a more useful checklist for this type of study and this has been uploaded to the extended data (Appendix 2). (NB please note this may take 24 hours to appear on the repository). This also meets CREDES criteria and is available on request if you think this would be helpful.*

 2. Ethics section: The ethical approval reference (LSHTM REC 33189) is provided, but the protocol does not address compensation or data privacy for participants from outside the UK, which is relevant given the recruitment strategy across multiple regions, including sub-Saharan Africa.

*We are not providing compensation for panellists in this Delphi study. We are recruiting professionals and asking for a relatively short amount of time, 4-5 x 20-minute surveys over a 6-month period. This is in line with other published Delphi studies. Data is managed in line with institutional policies and is clearly documented in line 421.*

 3. WhatsApp reminders: If you have agreed to receive a phone reminder, the study facilitator will send you a WhatsApp message to remind you. If you want to use a commercial, non-GDPR-compliant messaging platform to contact the participants, you need to have a good reason and get the go-ahead from the ethics committee, which is not mentioned.

*The decision for use of WhatsApp reminders has been approved by the ethics committee. To be clear, no data will be collected via WhatsApp it is used as an alternative method of professional communication to complement e-mail reminders and to ensure our study is accessible to busy healthcare professionals in multiple settings. We have updated this in line 364.* “WhatsApp is widely used to communicate between healthcare professionals globally as an adjunct to email communication ^25–27^. It is not country specific and is end-to-end encrypted. The use of WhatsApp in addition to email as means of professional communication is equitable and is likely to increase participation ^26^.”

# Reviewer report: Reviewer response for version 1

**DOI:** 10.21956/wellcomeopenres.28878.r155499

**Published:** 2026-05-27

**Authors:** Tarun Narang

**Affiliations:** 1 Post Graduate Institute of Medical Education and Research, Chandigarh, Punjab, India

This is a well-designed, highly necessary, and methodologically sound study protocol targeting a neglected dermatological manifestation in a vulnerable patient population (perinatally acquired HIV). However a few issues need to be taken care of 

**Definition of Attrition Bias Safeguards: **While the protocol states non-responders in one round can rejoin future rounds, dropping below a baseline number of active participants can heavily skew consensus. The authors should declare a minimum acceptable response rate per round (e.g., 70% or at least 15 active panelists) to validate continuous data.

**Consensus Threshold Discrepancy: **In the abstract and text, consensus is defined as >60% (moderate consensus) for individual items and ≥80% (strong consensus) for the criteria as a whole. However, the "Criteria for Termination" section mentions that surveys will run iteratively until strong consensus (>80%) is reached on the draft criteria. The text should be harmonized to clarify if an individual item with only 63% agreement will terminate the round or face further iteration.

 Clarify Region Representation: Ensure that the rule to include at least 4 WHO regions is strictly tied to a minimum number of representatives per region so that one or two regions do not disproportionately drown out low-resource diagnostic realities.

Is the study design appropriate for the research question?

Yes

Is the rationale for, and objectives of, the study clearly described?

Yes

Are sufficient details of the methods provided to allow replication by others?

Yes

Are the datasets clearly presented in a useable and accessible format?

Not applicable

Reviewer Expertise:

Dermatology , Tropical skin infections

I confirm that I have read this submission and believe that I have an appropriate level of expertise to confirm that it is of an acceptable scientific standard, however I have significant reservations, as outlined above.
